# Tofacitinib as a Promising Therapeutic Option in Refractory Autoimmune-Mediated Vascular and Sclera Inflammation

**DOI:** 10.31138/mjr.20230929.taa

**Published:** 2023-09-29

**Authors:** Theodoros Dimitroulas

**Affiliations:** 4^th^ Department of Internal Medicine, Hippokration Hospital, School of Medicine, Aristotle University Thessaloniki, Greece

**Keywords:** JAK inhibitors, tofacitinib, autoimmune scleritis, Takayasu

Janus Kinase (JAK) inhibitors represent a relatively novel class of oral immunomodulatory agents approved for several immune mediated diseases such as rheumatoid arthritis, the spondyloarthropathies, and atopic dermatitis, all of which are characterised by systemic and/or local upregulation of inflammatory cytokines. Given that the JAK pathway constitutes a pivotal regulator of pro-inflammatory signal transduction, JAK inhibitors are considered to ameliorate the effects of several cytokines and provide a broader level of control in systemic auto-immune diseases beyond inflammatory arthritides.^1^ The latter is reflected in the growing number of studies exploring the efficacy of JAK inhibitors in new indications such as systemic lupus erythematosus or dermatomyositis.^2,3^

In the current issue of the Mediterranean Journal of Rheumatology, two case series of successful treatment with tofacitinib - a non-selective JAK1/2/3 inhibitor – administered “off-label” in refractory cases of Takayasu arteritis^4^ and non-infectious scleritis^5^ are presented. Takayasu arteritis is chronic systemic inflammatory condition characterized by vascular granulomatous inflammation leading to progressive intimal fibrosis, vessel-wall remodelling, and eventually stenosis and aneurysm formation in large vessels. Takayasu arteritis is associated with severe (cardio)vascular morbidity and mortality due to suboptimal control of vascular inflammation, the relapsing pattern of the disease and the adverse consequences of high cumulative steroid doses, which currently represent the cornerstone of treatment.^6^ More importantly, the lack of sufficient evidence in randomised control trials supporting the administration of conventional and biologic disease modifying drugs which are commonly used in daily practice highlights the unmet therapeutic needs in this condition.^7^

A plethora of innate and adaptive immune cells contribute to the pathogenic process of vascular remodelling in Takayasu arteritis. For example, enriched interferon-α and interferon-γ signatures have been reported in both residual CD4 and CD8 T-cells whilst interleukins −6 and −12 are aberrantly expressed by macrophages and monocytes in vascular lesions of patients suffering from the disease.^8^ Taking into account that the synthesis of each cytokine is driven by the JAK pathway, it is not surprising that tofacitinib reduced cytokine production and inhibited both T-cell and macrophage activation in a mouse model of large vessel vasculitis.^9^ Such data indicate that tofacitinib may be useful for the treatment of Takayasu disease, particularly in cases unresponsive to conventional treatment. Prakashini MV et al.^4^ report 8 cases of refractory Takayasu arteritis effectively controlled after the administration of tofacitinib 5mg bd. This is in line with other observations in the whole spectrum of large vessels vasculitis.^10^ suggesting that multicytokine blockade through JAK inhibition may be a promising option for the effective suppression of vascular inflammation in these conditions.^11^

Non-infectious inflammatory scleritis is an inflammatory ocular disease occurring as idiopathic or in the context of systemic inflammatory diseases. Although biologic drugs, namely, adalimumab, infliximab, and rituximab have been validated as targeted therapies in individuals suffering from systemic disorders, relapse may occur and lead to vision-threatening ocular complications. Dey at al.^5^ present three treatment-resistant cases of bilateral idiopathic scleritis in which remission was achieved after treatment with tofacitinib.

The pathophysiology of non-infectious scleritis remains largely unknown but immune activation of different cells populations including macrophages, Th1 and Th17 T-cells subsets as well as B- cells are pivotal drivers of the residual inflammatory process in sclera tissue.^12^ In this regard, tofacitinib may exert anti-inflammatory properties in both the ocular surface and intraocular inflammation by blocking the development and differentiation of TH1 cells and reducing the production of interferon-1 as demonstrated in animal models of experimental autoimmune uveitis.^13^ Similar observations have been reported with upadacitinib, a selective JAK-1 Inhibitor.^14^ Such experimental findings have been confirmed in a number of cases and short series describing the beneficial effects of JAK inhibition in patients suffering from a broad spectrum of inflammatory eye disorders.^15^

Despite the expanding use of JAK inhibitors across different medical specialties several safety concerns have been raised regarding herpes zoster infections, cardiovascular, and thromboembolic side effects as well as cancer occurrence.^16,17^ However, the complexity of immune-mediated diseases such as Takayasu arteritis and inflammatory scleritis in which numerous cytokines and various cell types interact with each other suggest that inhibition of multiple cytokine signalling pathways with tofacitinib rather that single cytokine blockade may be clinically valuable. Data from randomised trials and prospective studies will provide further evidence for the efficacy and safety of JAK inhibitors as their use is expanding in a diverse range of indications.
